# Thermogravimetric and calorimetric dataset for extra heavy crude asphaltenes obtained by electrodeposition in the presence of magnetic field and magnetic nanoparticles

**DOI:** 10.1016/j.dib.2020.105875

**Published:** 2020-06-18

**Authors:** Manuel Roa, Ivan Amaya, Rodrigo Correa

**Affiliations:** a Universidad Industrial de Santander, Bucaramanga, Colombia; b Tecnologico de Monterrey, Monterrey, Mexico

**Keywords:** Thermogravimetry (TGA), Differential scanning calorimetry (DSC), Asphaltene, Electrodeposition, Extra heavy oil, Nanoparticles

## Abstract

A better understanding of the behavior of asphaltenes is paramount for improving processes related to heavy crude oil, such as transport. Previous research has studied its aggregation [1], as well as its average chemical structure [2]. But, of course, this expands beyond the realm of oil, as other applications are affected by asphaltenes [3]. This work presents the collected data from electrodeposited asphaltenes. We used an H-type cell, with a capacity of 300 ml, and a bridge length of 30 cm. A constant voltage of 300 Vs (DC) was applied to steel electrodes of 3 cm × 9 cm. The generated electric field was of 1000 V/m, (*E* = Voltage (V)/distance (m)). The previously described assembly was modified, adding a dynamic magnetic field of 4 mT and 1% w/w of magnetic nanoparticles. Afterward, we analyzed deposits at the anode and cathode using the DSC-TGA Netzsch 449 F1 equipment. Through it, we gathered the calorimetric (DSC) and thermogravimetric (TGA) data. Moreover, the Proteus analysis software was used to generate DTGA data from the TGA values. The same procedure and analysis were repeated for asphaltenes of the same oil but obtained through precipitation with heptane. Our data may pave the road for research seeking to improve the extraction, transport and refining of heavy crudes. The reason: asphaltenes are responsible for the high viscosity of hydrocarbons. So, thermal processes are customary. Our thermogravimetric data may prove fruitful in the improvement and development of said processes.

**Specifications Table**

**Table:** 

**Subject**	Energy (General)
**Specific subject area**	Thermogravimetry and calorimetry of hydrocarbons
**Type of data**	TableGraph
**How data were acquired**	Thermogravimetry (TGA), differential scanning calorimetry (DSC), Instruments: Netzsch 449 F1Software: Proteus
**Data format**	Raw
**Parameters for data collection**	Electrodeposition 1:1000 V/mElectrodeposition 2:1000 V/m and 4mTElectrodeposition 3: 1000 V/m, 4mT and 1% w/w of magnetic nanoparticlesTGA and DSC data were obtained with a heating rate of 5 °C/min from 30 °C to 1000 °C
**Description of data collection**	Samples were obtained from electrodeposition processes. Asphaltenes were obtained from both, anode and cathode. The TGA and DSC data were obtained with the Netzsch 449 F1 equipment, considering a heating rate of 5 °C/min from 30 °C to 1000 °C. DTGA data was collected using the Proteus program.
**Data source location**	Institution: Universidad Industrial de SantanderCity/Town/Region: Bucaramanga/SantanderCountry: Colombia
**Data accessibility**	Repository name: Mendeley DataData identification number: 10.17632/8zv4yw4wc9.1Direct URL to data: https://data.mendeley.com/datasets/8zv4yw4wc9/1

**Value of the Data**

• Knowing the thermogravimetric behavior and the thermal transitions of asphaltenes will allow to predict and optimize its processing, either with the application of current thermal technologies, or through the development of new ones. This is required as asphaltenes lead to high viscosity in heavy crude oils, affecting the oil and energy industry.
• This article can benefit different kinds of researchers. Those interested in chemical structure could use it to improve or test their structural models. Similarly, researchers on oil refining may use it to design better equipment and thermal procedures. Moreover, it can improve the development of additives commonly used in heavy crude oil extraction and transportation.
• The data in this article includes information about mass losses and about thermal transitions. So, whenever planning an experiment with asphaltenes, at a certain temperature, data can be consulted to know if any of those phenomena manifest.
• When transporting crude oil, asphaltenes are electrodeposited in the walls of pipelines. This leads to large blockages and high costs. The data in this article may aid in developing and implementing thermal treatments for eliminating such blockages, without having to change any tube section. Such data can also help complement other data about asphaltenes, such as the one presented in [4] and in [5].

## Data description

This work presents thermogravimetric datasets of asphaltenes present in heavy crude oils, under different experimental conditions. We consider experiments where the electrodeposition is performed using an H-type cell, with a capacity of 300 ml and a bridge length of 30 cm. A constant voltage of 300  V (DC) is applied to a couple of steel electrodes of 3 cm × 9 cm. This generates an electric field of 1000 V/m, (*E*= Voltage (V)/distance (m)). A dynamic magnetic field of 4mT is also considered for some experiments.

Table 1 summarizes the experimental conditions for the information presented in tables within the dataset. All tests are carried out for samples of Colombian extra heavy crude between 30 °C and 1000 °C, with increments of 5 °C/min. Also, the first table contains asphaltene data for the control group: heavy crude oil processed with heptane. Therefore, such a sample does not undergo electrodeposition. After that, the dataset presents information in four pairs of tables, where the first one contains data from the anode while the other has the data for the cathode. So, the first experiment (Tables 2 and 3) deals with the effect of the electric field. Afterward, combined effects are displayed. On one hand, Tables 4 and 5 present the effect of including a magnetic field of 4 mT. On the other, Tables 6 and 7 show the effect of adding 1% w/w of carbon-nanomagnetite (CMG). Information from tables wrap up by presenting the total combined effect (electric and magnetic fields plus CMG) in Tables 8 and 9. It is important to highlight that the meaning of each column header is given in Table 2.

**Table 1:** Summary of experimental conditions for each dataset shown in tables. CMG: Carbon-nanomagnetite.

Table	Experimental conditions	Electrode
1	Control group: Heavy crude oil + heptane	–
2	Electric field (1000 V/m)	Anode
3	Electric field (1000 V/m)	Cathode
4	Electric field (1000 V/m) + magnetic field (4 mT)	Anode
5	Electric field (1000 V/m) + magnetic field (4 mT)	Cathode
6	Electric field (1000 V/m) + CMG (1% w/w)	Anode
7	Electric field (1000 V/m) + CMG (1% w/w)	Cathode
8	Electric field (1000 V/m) + magnetic field (4 mT) + CMG (1% w/w)	Anode
9	Electric field (1000 V/m) + magnetic field (4 mT) + CMG (1% w/w)	Cathode

**Table 2:** Description of the column headers found in the tables within the dataset.

Column name	Description of the data
Temperature (°C)	Temperature of the sample at the time the measurement is taken
Time (min)	Time elapsed between the start of the experiment and the moment when the measurement is taken
TG (%)	Percentage of mass remaining in the sample when the measurement is taken
DSC (uV/mg)	Differential Scanning Calorimetry data corresponding at the time when the measurement is taken
DTG (%/min)	Value of the derivative of TG w.r.t. time when the measurement is taken

**Table 3:** Summary of experimental conditions for each dataset shown in figures. CMG: Carbon-nanomagnetite.

Figure	Experimental conditions	Electrode	Property
1	Control group: Heavy crude oil + heptane	–	TGA
2	Control group: Heavy crude oil + heptane	–	DSC
3	Control group: Heavy crude oil + heptane	–	DTGA
4	Electric field (1000 V/m)	Anode	TGA
5	Electric field (1000 V/m)	Anode	DSC
6	Electric field (1000 V/m)	Anode	DTGA
7	Electric field (1000 V/m)	Cathode	TGA
8	Electric field (1000 V/m)	Cathode	DSC
9	Electric field (1000 V/m)	Cathode	DTGA
10	Electric field (1000 V/m) + magnetic field (4 mT)	Anode	TGA
11	Electric field (1000 V/m) + magnetic field (4 mT)	Anode	DSC
12	Electric field (1000 V/m) + magnetic field (4 mT)	Anode	DTGA
13	Electric field (1000 V/m) + magnetic field (4 mT)	Cathode	TGA
14	Electric field (1000 V/m) + magnetic field (4 mT)	Cathode	DSC
15	Electric field (1000 V/m) + magnetic field (4 mT)	Cathode	DTGA
16	Electric field (1000 V/m) + nanoparticles (1% w/w)	Anode	TGA
17	Electric field (1000 V/m) + nanoparticles (1% w/w)	Anode	DSC
18	Electric field (1000 V/m) + nanoparticles (1% w/w)	Anode	DTGA
19	Electric field (1000 V/m) + nanoparticles (1% w/w)	Cathode	TGA
20	Electric field (1000 V/m) + nanoparticles (1% w/w)	Cathode	DSC
21	Electric field (1000 V/m) + nanoparticles (1% w/w)	Cathode	DTGA
22	Electric field (1000 V/m) + magnetic field (4 mT) + nanoparticles (1% w/w)	Anode	TGA
23	Electric field (1000 V/m) + magnetic field (4 mT) + nanoparticles (1% w/w)	Anode	DSC
24	Electric field (1000 V/m) + magnetic field (4 mT) + nanoparticles (1% w/w)	Anode	DTGA
25	Electric field (1000 V/m) + magnetic field (4 mT) + nanoparticles (1% w/w)	Cathode	TGA
26	Electric field (1000 V/m) + magnetic field (4 mT) + nanoparticles (1% w/w)	Cathode	DSC
27	Electric field (1000 V/m) + magnetic field (4 mT) + nanoparticles (1% w/w)	Cathode	DTGA

In a similar fashion, Table 3 summarizes the experimental conditions associated to the datasets presented in the figures. In short: each experimental condition from Table 1 has three figures associated, one for TGA, one for DSC, and one for DTGA. This way, the triplet given by Figs. 1–3 show the behavior of the control group, whilst Figs. 25–27 do so for the asphaltene gathered from the cathode when considering the total combined effect (electric and magnetic field plus CMG), which is the final experimental condition from Table 1.

## Experimental design, materials and methods

Asphaltene data were collected after electrodeposition processes that connected to a precipitation with heptane. Electrodepositions were carried out in an H Pyrex cell, with a capacity of 300 ml, and with a bridge length of 30 cm. A constant voltage of 300  V (DC) was applied to 3 cm × 9 steel electrodes, which generated an electric field of 1000 V/m, (*E* = Voltage (V)/distance (m)). Some experiments considered the addition of 1% w/w of carbon-nanomagnetite (CMG) compound within the extra heavy crude, as well as the effect of a magnetic field applied with a 4 mT dynamic magnet. Experiments were run for combinations of these conditions, and deposits at the anode and cathode were collected.

The experimental procedure for the thermoanalytic characterization (TGA and DSC) started with the purification of asphaltene samples through several precipitations with heptane. The samples with the asphaltenes electrodeposited in the steel electrodes were removed by making them soluble with toluene and then precipitating them with heptane. Each asphaltene sample was subject to a thermogravimetric analysis (TGA) and to a differential scanning calorimetry (DSC) in the Netzsch 449 F1 equipment. To this end, we used alumina crucibles of 146 mg. For each analysis, we used 10 mg of asphaltenes. The system was preheated at 30 °C and it was purged with nitrogen. Throughout the TGA, samples were processed at a heating rate of 5 °C/min, starting at 30 °C and going up to 1000 °C, while also keeping an inert nitrogen atmosphere. After compiling the thermogravimetric curves (TGA), their first derivative (DTGA) was calculated through the Proteus software, which comes with the equipment. Conversely, we used samples of 25 mg for generating the DSC curves, set on aluminum sample holders, and at a heating rate of 5 °C/min.

## Declaration of Competing Interest

The authors declare that they have no known competing financial interests or personal relationships which have, or could be perceived to have, influenced the work reported in this article.

## Appendix Supplementary materials

Image, application 1
